# Epidemiological Trends and Factors Associated With the Morbidity Rate of Gonorrhea: A CDC-WONDER Database Analysis

**DOI:** 10.7759/cureus.42981

**Published:** 2023-08-05

**Authors:** Micheal K Akinboro, John Mmaduabuchi, Papa Kwame Antwi Beeko, Obinna F Egwuonwu, Oluwabukola P Oluwalade, Ngozi T Akueme, Blessing O Iyioku, Okelue E Okobi, Etakewen Paul Oghenetega

**Affiliations:** 1 Epidemiology and Biostatistics, Texas A&M University School of Public Health, College Station, USA; 2 Emergency Department, Eastway Medical Center and Urgent Care, Charlotte, USA; 3 General Medicine, Suhum Government Hospital, Suhum, GHA; 4 Family Medicine, University of Nigeria Teaching Hospital, Enugu, NGA; 5 Family Medicine, All Saints University School of Medicine, Roseau, DMA; 6 Dermatology, University of Medical Sciences, Ondo City, NGA; 7 Dentistry, Oral Health Africa Initiative, Alberta, CAN; 8 Family Medicine, Larkin Community Hospital Palm Springs Campus, Miami, USA; 9 Family Medicine, Medficient Health Systems, Laurel, USA; 10 Family Medicine, Lakeside Medical Center, Belle Glade, USA; 11 Internal Medicine, Milton Keynes University Hospital, Buckinghamshire, GBR

**Keywords:** us, usa, united states, epidemiological trend analysis, morbidity rate, cdc-wonder, gonorrhea

## Abstract

Background and objective: Gonorrhea is a prevalent sexually transmitted illness caused by the bacteria Neisseria gonorrhoeae, leading to serious health consequences such as pelvic inflammatory disease (PID), infertility, ectopic pregnancy, and increased susceptibility to HIV infection. Despite advancements in prevention and treatment, gonorrhea remains a significant public health problem in the United States (US) due to its widespread incidence, potential consequences, and the growth of antibiotic resistance. This study investigates the epidemiological trends and morbidity rates of gonorrhea using the Centers for Disease Control and Prevention's (CDC) Wide-ranging Online Data for Epidemiologic Research (WONDER) database. The aim is to identify temporal patterns, demographic characteristics, and notable changes in gonorrhea epidemiology to inform targeted therapies and interventions.

Methods: The CDC WONDER database, which provides extensive national and state-level data on reported causes of death in the United States, was utilized for this study. We examined the developments in gonorrhea morbidity rates over time, identified demographic differences based on age, gender, and race/ethnicity, and analyzed the disease's regional distribution through a systematic analysis of the database. Aggregate data for selected time periods (1996-2014) were summarized using the morbidity rate per 100,000 people and the total number of cases across the years.

Results: This database analysis identified a total of 6,454,097 individuals diagnosed with gonorrhea between 1996 and 2014. The calculated total morbidity rate during this period was 115.4 per 100,000 individuals. The highest morbidity rates were observed in the years 1999 (129.2 per 100,000 people), 1998 (129.1 per 100,000 people), and 2001 (126.8 per 100,000 people), respectively. The District of Columbia reported the highest morbidity rate (478.25 per 100,000 people). In males, the overall morbidity rate over the years was reported to be comparable to females (114 per 100,000 people and 116.3 per 100,000 people, respectively). The analysis revealed consistently higher morbidity rates among individuals aged between 19 and 24 years (525.2 per 100,000 people). Moreover, black or African American individuals consistently exhibited higher morbidity rates (506.1 per 100,000 people) compared to white individuals (16.1 per 100,000 people).

Conclusion: The analysis of gonorrhea cases between 1996 and 2014 revealed that the highest rates occurred during specific years, with a particular concentration observed in the District of Columbia. Additionally, certain demographic groups, such as individuals aged 19-24 and the black or African American population, consistently exhibited higher morbidity rates compared to others. These findings emphasize the importance of targeted interventions to address the observed temporal patterns and demographic disparities, in order to effectively combat the spread of gonorrhea.

## Introduction

Gonorrhea is a highly prevalent sexually transmitted infection caused by the bacterium Neisseria gonorrhoeae, affecting people globally. It can lead to serious health complications, including pelvic inflammatory disease (PID) in women, infertility, ectopic pregnancy, and an increased risk of human immunodeficiency virus (HIV) infection [1-3]. In the United States (US), despite advancements in prevention and treatment, gonorrhea remains a significant public health problem due to its widespread incidence, potential consequences, and the emergence of antibiotic resistance [4-5].

Gonorrhea poses substantial challenges to public health in the US. In recent years, there has been a concerning increase in antibiotic-resistant strains of Neisseria gonorrhoeae, complicating treatment and control efforts [6-13]. Additionally, certain populations, such as adolescents (10-19 years) and young adults (15-24 years), have been disproportionately affected by gonorrhea, with higher infection rates than other age groups [12]. The impact of gonorrhea extends beyond the individual level, as it imposes a considerable economic burden on healthcare systems due to the costs associated with testing, treatment, and management of its complications [13].

Understanding the epidemiological patterns and morbidity rates associated with gonorrhea is crucial for effectively addressing the burden of the disease and developing targeted interventions. Examining morbidity patterns over time can help identify changes in disease burden, detect outbreaks or epidemics, and evaluate the effectiveness of preventive and control measures [6]. According to the Centers for Disease Control and Prevention (CDC) surveillance report in 2018, there were 583,405 recorded cases of gonorrhea in the US, with a morbidity rate of 179.1 per 100,000 person [7].

The availability of extensive health datasets in recent years has facilitated in-depth investigations into the epidemiological patterns and morbidity rates of various health disorders, including sexually transmitted diseases (STDs). The CDC in the US maintains the CDC WONDER (Wide-ranging Online Data for Epidemiologic Research) Underlying Cause of Death Database, which provides national and state-level data on causes of mortality and morbidity, allowing valuable insights into the impact of gonorrhea on public health [8-10]. The morbidity of gonorrhea refers to its occurrence and impact within a community. Morbidity measurements shed light on the prevalence, distribution, and burden of gonorrhea cases, aiding in better understanding the public health implications and guiding intervention strategies [6,10].

By analyzing the CDC WONDER database, this study aims to contribute to understanding the epidemiology of gonorrhea in the US, providing valuable insights for public health practitioners and policymakers in developing targeted prevention strategies and interventions to address the ongoing challenges posed by this sexually transmitted infection. The study utilizes a retrospective observational design, analyzing aggregate data collected from the database between 1996 and 2014. By exploring temporal patterns in gonorrhea-related morbidity rates, including changes over time and variations among geographic areas, this study seeks to gain insights into the dynamics of the disease. Additionally, the study aims to identify demographic characteristics associated with a higher risk of gonorrhea-related morbidity, such as age, gender, and race, as well as any notable changes in gonorrhea epidemiology, such as shifts in age-specific morbidity rates, changes in comorbidity patterns, or emerging trends in geographic distribution.

## Materials and methods

Study sample

The CDC WONDER database was used to retrieve summarized morbidity data for gonorrhea. These reports were originally submitted to the "National Center for acquired immunodeficiency syndrome, viral hepatitis, STD, and tuberculosis prevention" (NCHHSTP) [8]. The focus of the query was individuals diagnosed with gonorrhea between 1964 and 2014. The database primarily provides morbidity data, including the number of cases and disease incidence rates categorized by year, type of STD, and geographical location. Additionally, the data were further categorized by variables such as gender, age group, race/ethnicity, and other relevant details [8]. Since the data within the database does not contain any identifiable information and is publicly available through a data use agreement with the National Center for Health Statistics, there was no need for ethical committee approval or informed consent. The analyses for this study were conducted between May 20, 2023, and June 10, 2023. 

Inclusion and exclusion criteria

The study included all the datasets obtained from the database for subjects diagnosed with gonorrhea between 1996 and 2014. No restrictions were placed on age, gender, or geographical location. The inclusion of all relevant cases during the specified time frame aimed to obtain a representative sample for analysis. However, the proportion of cases with unknown labels and suppressed data was excluded from the analysis.

Variables and measures

The primary outcome measure of interest was the morbidity rate of gonorrhea, calculated per 100,000 people. Additionally, trends in morbidity rates over the study period were examined to identify any significant changes or patterns. The analysis also focused on specific years with the highest morbidity rates to further explore temporal variations.

Statistical analysis

Aggregate data for selected time periods (1996-2014) and available patient characteristics were summarized using the morbidity rate per 100,000 person and the total number of deaths over the years were examined for all patients. Only patients with available data were included in each model, and effective sample sizes are presented in all tables and figures. No adjustments were made for multiple comparisons or unavailable data. Excel 2019 was used for all statistical analyses. Appropriate statistical methods were also employed to identify temporal trends and assess the significance of any observed variations in morbidity rates.

Ethical approval

The CDC WONDER dataset, provided by the CDC, is a publicly available database that we utilized for our analysis. Since this dataset consists of publicly available and de-identified information, it does not contain any identifiable information about individuals. Therefore, according to ethical research guidelines, analyzing such publicly available data using a secondary data analysis technique does not require Institutional Review Board (IRB) approval. Our study adhered to these guidelines by exclusively utilizing the de-identified and publicly available CDC WONDER dataset. The CDC maintains proper ethical standards for data collection, and their IRBs are constituted in accordance with the regulations outlined in 45 CFR part 46 and 21 CFR part 56.

## Results

We collected data from the database on 6,454,097 individuals diagnosed with gonorrhea between 1996 and 2014. The overall morbidity rate during this period was found to be 115.4 per 100,000 people. The highest morbidity rates were observed in the years 1999 (129.2 per 100,000 people), 1998 (129.1 per 100,000 people), and 2001 (126.8 per 100,000 people), respectively (Table 1).

**Table 1:** Morbidity rate trend year wise for demographic characteristics (1996-2014)

		Morbidity rate per 100,000																		
	Variables	1996	1997	1998	1999	2000	2001	2002	2003	2004	2005	2006	2007	2008	2009	2010	2011	2012	2013	2014
	Total number of cases	327723	326971	356107	360598	362920	361758	351836	335104	330132	339593	358366	355991	336742	301174	309341	321849	334826	333004	350062
	Total Population	269M	273M	276M	279M	282M	285M	288M	291M	294M	296M	299M	302M	304M	307M	309M	312M	314M	316M	316M
	Overall morbidity rate	121.7	119.9	129.1	129.2	128.6	126.8	122.0	115.2	112.4	114.6	119.7	118.0	110.8	98.1	100.2	103.3	106.7	105.3	110.7
STD region	West	51.4	49.83	52.6	51.2	57.18	60.18	62.78	63.23	71.77	80.48	81.5	73.19	60.71	52.72	58.38	61.41	72.57	82.72	101.14
	Midwest	121.85	120.06	136.99	137.01	145.66	142.46	142.25	135.78	133.69	138.6	136.44	138.97	129.81	115.98	108.34	110.84	114.3	108.23	106.56
	Northeast	88.29	85.88	84.74	89.29	88.47	97.1	93.63	90.84	80.32	74.6	73.72	77.22	70.91	67.05	77.35	85.44	92.21	85.25	84.71
	South	183.94	182.41	196.07	194.8	184.12	174.74	161.77	148.13	141.62	141.79	156.89	154.09	150.72	131.37	132.72	133.76	130.54	127.37	131.44
Gender	Female	117.3	118.6	128.0	126.2	126.3	126.6	122.5	117.9	115.4	118.0	123.1	122.6	118.5	104.5	105.6	108.0	107.9	101.7	101.3
	Male	126.2	121.2	130.0	132.1	130.4	126.5	121.1	111.9	108.8	110.4	115.6	112.8	102.1	91.0	93.9	97.8	105.0	108.7	120.1
Race	White	18.8	18.5	20.5	20.7	21.4	22.0	23.5	24.7	25.4	26.8	28.0	27.0	24.1	21.2	23.4	25.1	29.7	32.4	36.8
	Black or African American	594.0	579.5	637.4	635.1	612.4	577.3	548.4	499.6	483.5	477.6	504.9	515.2	489.0	431.1	426.2	421.9	417.4	384.5	381.9
	Asian or Pacific Islander	11.6	12.3	14.2	14.9	20.7	18.6	16.3	16.8	15.9	20.4	15.7	14.5	15.4	13.7	14.4	14.7	17.2	17.9	20.2
	American Indian or Alaska Native	77.4	71.4	81.4	76.1	75.5	76.4	85.2	77.6	89.2	100.2	105.2	82.8	83.9	87.0	107.4	114.4	123.2	135.5	157.7
	Hispanic	46.5	45.8	50.4	50.3	53.5	53.1	53.0	53.4	53.3	55.6	57.7	52.9	50.9	44.6	47.9	52.2	59.6	64.1	71.5
Age data	0-14 years	12.5	11.3	12.1	11.7	11.5	11.3	10.3	9.2	8.1	7.8	7.8	7.3	6.7	5.3	5.4	5.7	5.6	4.7	4.4
	15-19 years	520.5	498.2	527.7	517.3	503.3	494.9	469.5	438.3	419.7	429.7	450.7	457.6	451.2	403.9	400.4	407.2	381.8	340.7	323.6
	19-24 years	520.3	520.7	576.5	580.5	591.7	585.2	552.4	512.5	489.4	502.7	523.3	529.8	516.4	467.3	489.3	504.3	510.2	495.9	509.8
	25-29 years	244.4	246.9	279.0	283.7	292.5	292.6	290.4	278.3	278.9	283.6	297.2	290.4	265.6	230.0	241.2	250.2	273.1	287.8	322.5
	30-34 years	140.3	139.9	152.4	155.4	155.6	155.3	152.0	146.5	147.6	148.9	158.1	154.6	140.6	123.7	127.3	132.4	150.3	160.2	180.6
	35-39 years	88.9	87.6	98.6	101.2	100.5	97.0	97.1	93.8	93.5	94.5	97.1	89.9	78.0	68.0	68.2	72.0	83.1	92.0	106.1
	40+ years	19.9	20.1	22.2	23.3	22.7	22.0	22.0	21.6	22.6	22.9	23.9	22.2	18.4	15.0	15.1	16.2	18.5	20.4	22.5

Morbidity rate trend based on location

The study observed variations in the morbidity rate for gonorrhea in different locations over a specific time period. Table 2 provides data on the trend of morbidity rates based on location, including the nine regions mentioned in the morbidity and mortality weekly report (MMWR), the ten regions of the health and human services (HHS), and the top five US states, covering the years 1996 to 2014.

**Table 2:** Morbidity rate trend based on location (1996-2014) *Top five selected states with the highest morbidity rate. HHS: Health and human services, MMWR: Morbidity and mortality weekly report. Alabama: AL, Alaska: AK, Arizona: AZ, Arkansas: AR, California: CA, Colorado: CO, Connecticut: CT, Delaware: DE, Florida: FL, Georgia: GA, Hawaii: HI, Idaho: ID, Illinois: IL, Indiana: IN, Iowa: IA, Kansas: KS, Kentucky: KY, Louisiana: LA, Maine: ME, Maryland: MD, Massachusetts: MA, Michigan: MI, Minnesota: MN, Mississippi: MS, Missouri: MO, Montana: MT, Nebraska: NE, Nevada: NV, New Hampshire: NH, New Jersey: NJ, New Mexico: NM, New York: NY, North Carolina: NC, North Dakota: ND, Ohio: OH, Oklahoma: OK, Oregon: OR, Pennsylvania: PA, Rhode Island: RI, South Carolina: SC, South Dakota: SD, Tennessee: TN, Texas: TX, Utah: UT, Vermont: VT, Virginia: VA, Washington: WA, West Virginia: WV, Wisconsin: WI, Wyoming: WY.

Variables	Region	Morbidity Rate per 100,000
MMWR region	Division 1: New England	44.77
	Division 2: Middle Atlantic	97.11
	Division 3: East North Central	144.75
	Division 4: West North Central	87.37
	Division 5: South Atlantic	153.02
	Division 6: East South Central	173.43
	Division 7: West South Central	146.22
	Division 8: Mountain	58.19
	Division 9: Pacific	69.49
HHS region	HHS Region #1 CT, ME, MA, NH, RI, VT	44.77
	HHS Region #2 NJ, NY	96.11
	HHS Region #3 DE, DC, MD, PA, VA, WV	117.82
	HHS Region #4 AL, FL, GA, KY, MS, NC, SC, TN	165.36
	HHS Region #5 IL, IN, MI, MN, OH, WI	136.1
	HHS Region #6 AR, LA, NM, OK, TX	142.23
	HHS Region #7 IA, KS, MO, NE	103.77
	HHS Region #8 CO, MT, ND, SD, UT, WY	41.37
	HHS Region #9 AZ, CA, HI, NV	76.75
	HHS Region #10 AK, ID, OR, WA	42.75
State*	District of Columbia	478.25
	Mississippi	258.07
	Louisiana	234.48
	South Carolina	222.95
	Alabama	218.18

**Figure 1:**
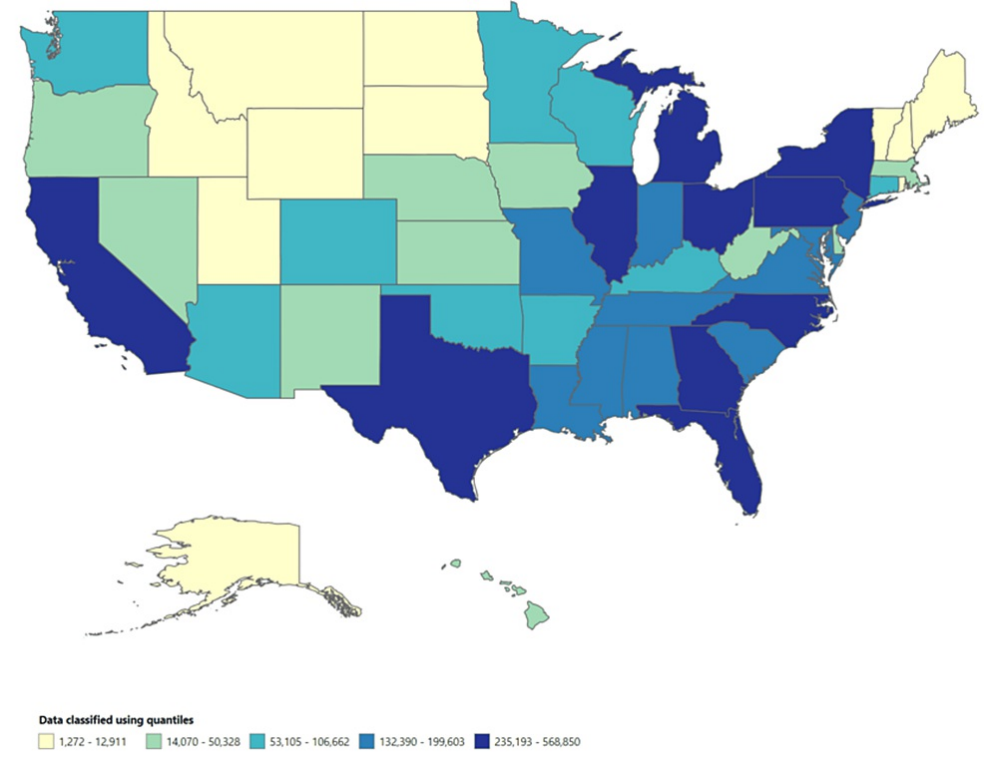
Total number of cases across the US (1996-2014)

**Figure 2:**
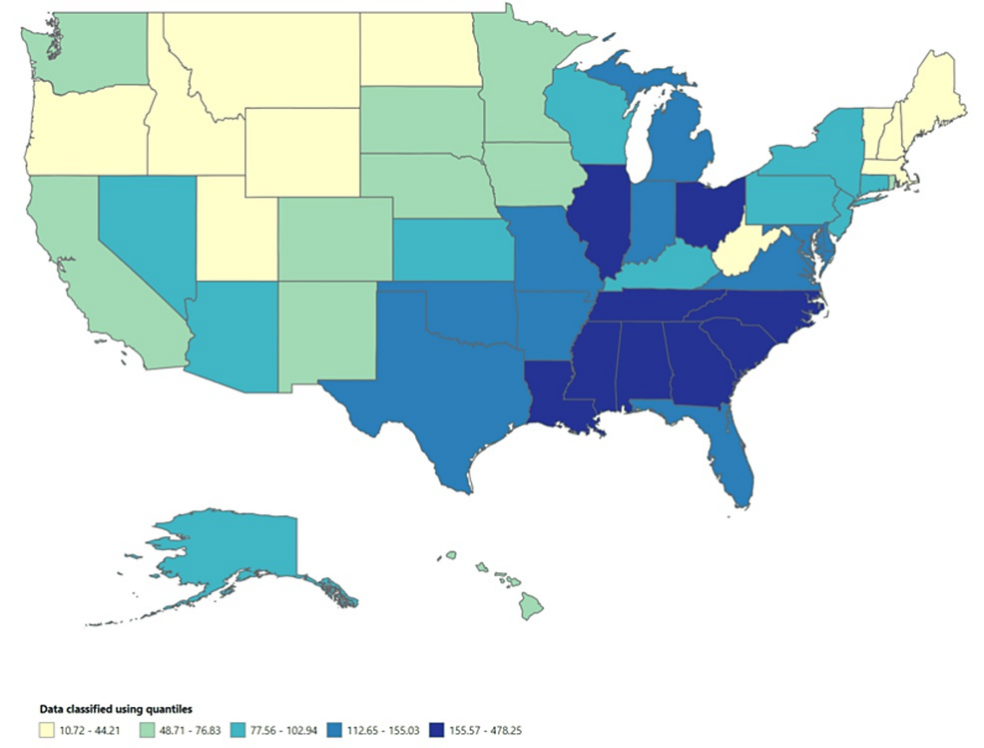
Morbidity rate across the US (1996-2014)

Throughout the study period, specific states consistently showed high morbidity rates for gonorrhea. The District of Columbia reported the highest rate at 478.25 per 100,000 people, followed by Mississippi at 258.07, Louisiana at 234.48, South Carolina at 222.95, and Alabama at 218.18 (Figures 1-2).

Among the nine MMWR regions, the South Atlantic region had the highest morbidity rate (153.02 per 100,000 people), followed by the East South Central region (173.43 per 100,000 people). In the HHS region, the highest morbidity rate was reported in HHS Region 4 (165.36 per 100,000 people). These areas exhibited notably higher morbidity rates, indicating a greater need for targeted interventions and public health initiatives. 

Demographics and gender

**Figure 3:**
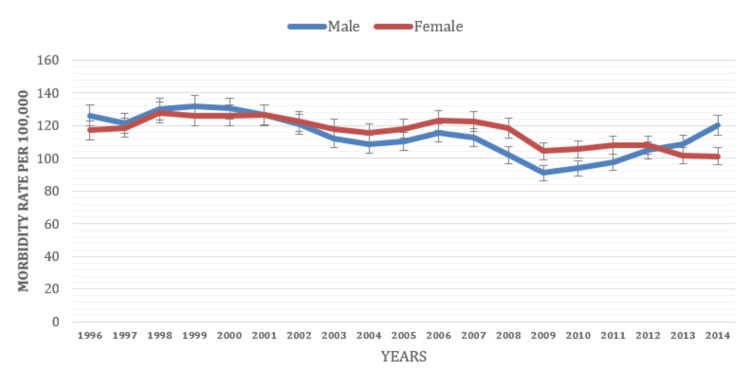
Morbidity rate based on gender classification (1996-2014)

The analysis of morbidity rate trends based on demographic characteristics yielded findings that indicated gender-based variations and explored potential factors associated with gonorrhea morbidity rates. The study examined the morbidity rates per 100,000 people for various demographic groups from 1996 to 2014. Gender-wise, the overall morbidity rate for males (114 per 100,000 people) was found to be comparable to that of females (116.3 per 100,000 people). Among males, the morbidity rate showed a gradual decrease from 1996 (126.2 per 100,000 people) to 2009 (91 per 100,000 people), followed by a slight increase until 2011 (97.8 per 100,000 people). Subsequently, it experienced a significant increase in 2014 (120 per 100,000 people). For females, the morbidity rate exhibited a fluctuating pattern, with a gradual decrease from 1996 (117.3 per 100,000 people) to 2009 (104.5 per 100,000 people). It remained relatively stable until 2011 (101.3 per 100,000 people) (Figure 3).

Age groups

**Figure 4:**
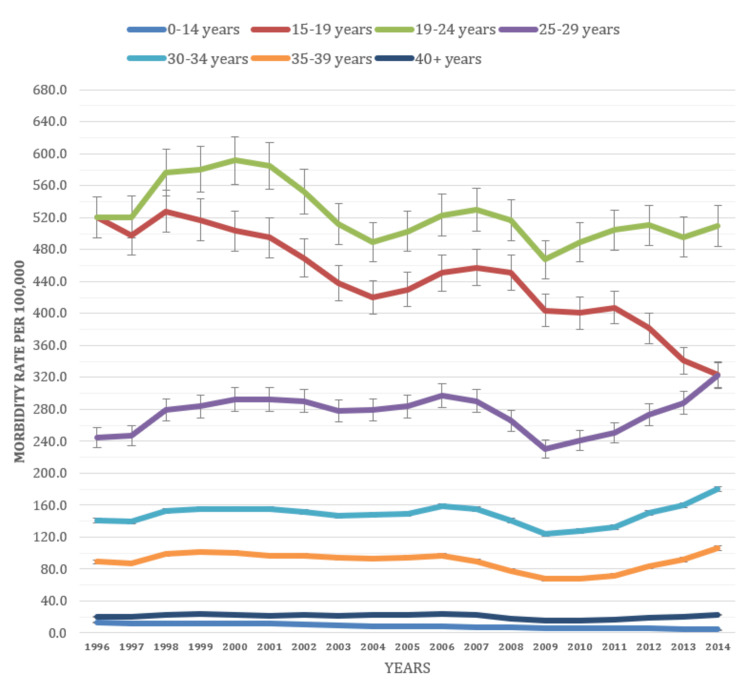
Morbidity rate age classification (1996-2014)

The study revealed significant variations in disease morbidity rates among different age groups. Notably, individuals aged between 19 and 24 years consistently displayed higher morbidity rates, with a rate of 525.2 per 100,000 people. Following closely were individuals aged between 15 and 19 years, with a rate of 444 per 100,000 people. Among adults aged between 30 and 34 years, there was a noteworthy increase in morbidity rates, rising from 140.4 per 100,000 people in 1996 to 180.6 per 100,000 people in 2014. Interestingly, older adults aged 40 years and above exhibited a different trend. Their morbidity rates steadily decreased from 1996 to 2014, indicating a positive trend in reducing morbidity rates in this age group (Figure 4).

**Figure 5:**
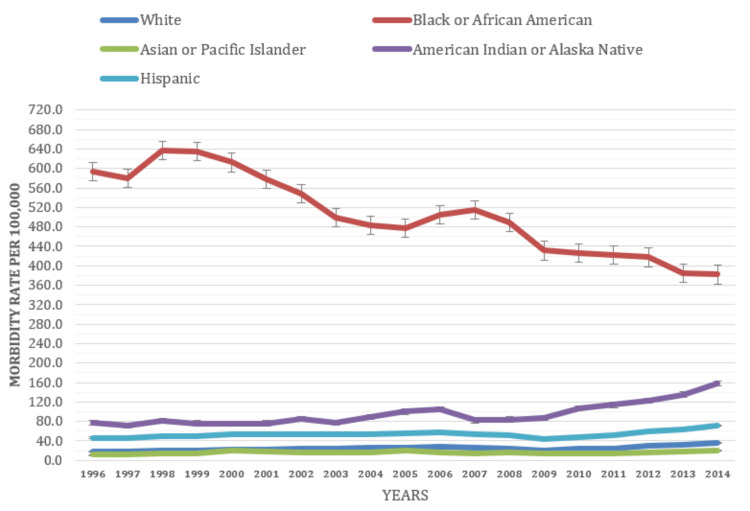
Morbidity rate based on race (1996-2014)

The overall morbidity rate for gonorrhea exhibited variations among different racial groups throughout the duration of the study. When examining the morbidity rates of gonorrhea by race, significant disparities in the prevalence of the disease were observed across various racial groups. The analysis revealed that individuals identifying as black or African American consistently had higher morbidity rates for gonorrhea (506.1 per 100,000 people) compared to those identifying as white (16.1 per 100,000 people), American Indian or Alaska Native (53.5 per 100,000 people), and Asian or Pacific Islander (95.1 per 100,000 people) (Figure 5).

Over the study period, the morbidity rate for gonorrhea among individuals of black or African American race exhibited a decreasing trend (1996: 594 per 100,000 people, 2014: 381.9). On the other hand, among individuals of American Indian or Alaska Native race, the gonorrhea morbidity rate demonstrated an increasing trend (1996: 77.4 per 100,000 people, 2014: 157.5). This indicates the necessity of targeted interventions and preventive measures within this racial group to alleviate the burden of gonorrhea.

## Discussion

The morbidity rate for gonorrhea during the study period was determined to be 115.4 per 100,000 people. This result aligns with previous research that reported similar findings [6,11-12]. The analysis aimed to examine how morbidity rates for gonorrhea varied across different geographical locations. Specific geographical locations, such as the District of Columbia (478.25 per 100,000 people) and Mississippi (258.07 per 100,000 people), consistently exhibited high morbidity rates for gonorrhea throughout the study period. Additionally, the South Atlantic region (153.02 per 100,000 people) had the highest morbidity rate among the nine MMWR regions. These findings generally support our results, although they contrast with a previously published surveillance report where the District of Columbia (611 per 100,000 people) reported a higher morbidity rate [7]. These findings emphasize the importance of considering location-specific factors when developing and implementing prevention and control methods. Understanding the locations with the highest morbidity rates can aid in resource allocation and the implementation of measures to limit transmission and improve access to healthcare services in those areas.

The study also examined the year-to-year trend of morbidity rates for demographic characteristics such as age, gender, and race. The findings revealed variations in morbidity rates across different demographic groups over time. The overall morbidity rate for males (114 per 100,000 people) was similar to that of females (116.3 per 100,000 people) over the years. The CDC case surveillance data also indicated that males had a higher morbidity rate than females [6,7]. The findings showed stable morbidity trends among both males and females, underscoring the need for prevention strategies and healthcare services targeting both genders.

Overall, the study's findings highlight the year-to-year changes in gonorrhea morbidity rates among various ethnic groups. These findings emphasize the importance of tailored interventions, preventive measures, and healthcare services for specific racial groups, particularly black or African American (506.1 per 100,000 people) and American Indian or Alaska Native (95.1 per 100,000 people), to effectively address the burden of gonorrhea and reduce its impact on affected populations. These findings are consistent with a previously published analysis that found Blacks had 7.7 times the rate of Whites [6,7,13-15]. Additionally, recognizing intersectionality and understanding the specific health needs of multiracial individuals is crucial for equitable healthcare delivery and addressing gonorrhea disparities.

Morbidity rates for gonorrhea varied significantly across different age groups. Adolescents (15-19 years) had a morbidity rate of 444 per 100,000 people, emphasizing the need for age-appropriate treatments and education programs to prevent and manage gonorrhea in this vulnerable demographic. Young adults (19-24 years) had the highest morbidity rate of 525.2 per 100,000 people, highlighting the importance of targeted awareness programs and accessible healthcare services for this age group. These findings align with previous CDC reports, although the morbidity rate for adolescents and young adults was reported to be higher in our study. Another study conducted in the US by Martinez et al. also revealed a higher morbidity rate, but the age trends were consistent with our findings, with the 19-24-year age group having the highest morbidity rate, followed by the 15-19-year age group [6,7,11-22]. Our findings were also consistent with another published study [16-19].

The declining morbidity rates among adults and older individuals underscore the need for targeted treatments that consider age-related characteristics, as well as appropriate healthcare services for early identification and management. Several factors may contribute to the observed temporal patterns in morbidity rates for demographic parameters. Changes in healthcare access, screening practices, public health initiatives, and overall population health behaviors can all influence reported morbidity rates. Additionally, when interpreting the findings, it is important to consider changes in data collection techniques and reporting systems between time periods and geographic regions.

While this database analysis provides valuable information about the geographic distribution of gonorrhea morbidity rates, several limitations must be acknowledged. Since many gonorrhea cases go undetected or unreported, morbidity estimates may not accurately reflect the disease prevalence. Furthermore, the reported statistics do not capture changes in morbidity rates based on socioeconomic class. Additionally, the database primarily focuses on reported cases of gonorrhea, potentially missing asymptomatic cases, or those treated by private healthcare practitioners. These limitations should be considered when interpreting the findings and developing actions based on them. Nevertheless, analyzing existing data can offer significant insights into the epidemiology of gonorrhea and guide public health initiatives to mitigate its impact.

Despite these limitations, the study design allowed for a comprehensive analysis of gonorrhea epidemiological trends and morbidity rates using a large-scale database, providing valuable insights into the impact of this STD on the population over an extended period of time. Overall, the year-by-year morbidity rate trends for demographic factors revealed diverse patterns. While some groups showed decreasing or stable morbidity rates over time, others exhibited oscillations or inconsistent patterns. These findings underscore the importance of considering demographic parameters when analyzing gonorrhea epidemiology and developing tailored prevention and management strategies. It should be noted that these findings are based on data available during the research period (1996-2014) and may not reflect more recent developments. Further study and analysis are necessary to gain a deeper understanding of the current morbidity rate patterns among various demographic groups.

## Conclusions

Based on the database analysis, it can be inferred that gonorrhea has been a significant public health concern between 1996 and 2014, with a total of 6,454,097 diagnosed cases. The highest morbidity rates were observed in specific years, particularly 1999, 1998, and 2001, indicating temporal variations in the prevalence of the disease. The District of Columbia stood out as having the highest morbidity rate, suggesting the presence of localized factors contributing to the spread of Gonorrhea. Furthermore, the analysis highlighted demographic inequalities in morbidity rates. Males and females had comparable overall rates, suggesting that both genders were equally susceptible to contracting the disease. However, individuals aged 19-24 consistently exhibited higher morbidity rates, indicating that this age group may engage in riskier behaviors or have greater exposure to infection. Additionally, the consistently higher morbidity rates among the black or African American population compared to the white population indicate underlying disparities that need to be addressed. These findings underscore the need for targeted interventions and preventive initiatives to tackle the observed temporal patterns, demographic inequalities, and geographic differences in gonorrhea cases. By understanding the trends and disparities, there is a need to address at-risk populations, raise awareness, improve access to healthcare, and implement strategies to reduce transmission rates.
